# VE-Albumin Core-Shell Nanoparticles for Paclitaxel Delivery to Treat MDR Breast Cancer

**DOI:** 10.3390/molecules23112760

**Published:** 2018-10-25

**Authors:** Bo Tang, Yu Qian, Yi Gou, Gang Cheng, Guihua Fang

**Affiliations:** 1 School of Pharmacy, Nantong University, 19 Qixiu Road, Nantong 226001, Jiangsu, China; tangbo@ntu.edu.cn (B.T.); syfsxyhh18@163.com (Y.Q.); gouyi@ntu.edu.cn (Y.G.); 2 School of Pharmacy, Shenyang Pharmaceutical University, 103 Wenhua Road, Shenhe District, Shenyang 110016, Liaoning, China; chenggang63@hotmail.com

**Keywords:** Vitamin E, albumin, core-shell nanoparticles, paclitaxel, multi-drug resistance, breast cancer

## Abstract

Multi-drug resistance (MDR) presents a serious problem in cancer chemotherapy. In this study, Vitamin E (VE)-Albumin core-shell nanoparticles were developed for paclitaxel (PTX) delivery to improve the chemotherapy efficacy in an MDR breast cancer model. The PTX-loaded VE-Albumin core-shell nanoparticles (PTX-VE NPs) had small particle sizes (about 100 nm), high drug entrapment efficiency (95.7%) and loading capacity (12.5%), and showed sustained release profiles, in vitro. Docking studies indicated that the hydrophobic interaction and hydrogen bonds play a significant role in the formation of the PTX-VE NPs. The results of confocal laser scanning microscopy analysis demonstrated that the cell uptake of PTX was significantly increased by the PTX-VE NPs, compared with the NPs without VE (PTX NPs). The PTX-VE NPs also exhibited stronger cytotoxicity, compared with PTX NPs with an increased accumulation of PTX in the MCF-7/ADR cells. Importantly, the PTX-VE NPs showed a higher anti-cancer efficacy in MCF-7/ADR tumor xenograft model than the PTX NPs and the PTX solutions. Overall, the VE-Albumin core-shell nanoparticles could be a promising nanocarrier for PTX delivery to improve the chemotherapeutic efficacy of MDR cancer.

## 1. Introduction

Multi-drug resistance (MDR) is a large obstacle to the success of cancer chemotherapy and is crucial to cancer metastasis and recovery [1]. The well-known P-glucoprotein (P-gp), an ATP-binding cassette transporter, which is over expressed on the surface membrane of cancer cells, is one of the major reasons for the cancer MDR [2]. Many P-gp substrates, such as paclitaxel, were expelled out of the cancer cells, resulting in the reduction of intracellular drug accumulation, thereby leading to the treatment failure [3]. Therefore, it is urgent to explore a more effective strategy for overcoming the cancer MDR. 

Paclitaxel (PTX), a water-insoluble compound, is used widely as a fist-line drug in clinical treatment against variety of cancers [4]. PTX is commonly formulated as Taxol^®^, which uses Cremophor EL and dehydrated ethanol (50:50, V/V) as delivery vehicles to enhance its solubility. But this formulation often causes side effects, such as hypersensitivity, neuropathy, and neurotoxicity, which associate highly with Cremophor EL [5]. To mitigate these side effects, Abraxane^®^, a new formulation, was developed by using the high affinity between paclitaxel and serum albumin to prepare paclitaxel/albumin nanocomplex [5]. This formulation was approved by Food and Drug Administration in 2005. However, its anticancer effect is still greatly affected by cancer MDR. 

P-gp inhibitors have been studied for over P-gp mediated drug efflux, such as verapamil, dexverapamil, and tariquidar [6]. These P-gp inhibitors have been evaluated in clinic, but have not exhibited a good improvement in the therapeutic efficiency. These failures were mainly ascribed to the undesired toxicities, which have urged us to seek for new, more effective compounds with low toxicity and fewer side effects. Vitamin E (VE) is a lipid-soluble antioxidant, which protects lipids and membranes from oxidative damage [7]. It was reported that VE were not only able to overcome MDR by inhibition of ATPase activity, but also did not consider the toxicity [8,9]. In addition, water insoluble anticancer drugs, like PTX, can be loaded well in the VE-based emulsion for parental delivery [10]. Therefore, the use of VE as a P-gp inhibitor will be an attractive candidate to overcome the cancer MDR. However, VE is water-insoluble, which affects its administration in clinic.

Nano-drug delivery systems have been extensively investigated for anti-cancer drug delivery [11,12,13,14,15,16,17,18,19,20,21]. The nanocarriers could not only increase the water solubility of drugs but circumvent the P-gp efflux pump with entering the cancer cells by an endocytosis process [22]. Moreover, the nanoparticles provided a promising strategy for co-delivery of multiple drugs, in a single carrier, to improve the therapeutic efficiency of cancers [23,24,25,26,27,28]. Various nanocarriers have been developed for the co-delivery of anti-cancer drugs and P-gp inhibitors, such as the co-delivery of paclitaxel and borneol in lipid-albumin nanocomplex [29,30,31], docetaxel and verapamil in polymeric micelles [32], and paclitaxel and curcumin in lipid-albumin hybrid nanoparticles [33]. Additionally, VE is a lipid-soluble oil, which could be well encapsulated by the albumin to fabricate the VE-Albumin core-shell nanoparticles, through a hydrophobic interaction; VE and albumin could also make interactions with PTX. These interactions are beneficial for increasing the PTX-loading efficiency. Therefore, we speculate that co-delivery of the PTX and the VE with the VE-Albumin core-shell nanoparticles could improve the therapeutic efficiency of PTX against MDR cancers.

In this study, bovine serum albumin was used as a carrier to fabricate the VE-albumin core-shell nanoparticles co-delivery of the PTX and the VE. VE as the oil core of nanoparticles, not only increases the PTX-loading efficiency but also overcomes the P-gp-mediated drug efflux. The physicochemical properties and in vitro release were characterized. The cytotoxicity and cellular uptake were also investigated. Moreover, the anti-cancer effect was evaluated in breast cancer xenografts, in mice. It was speculated that the VE-albumin core-shell nanoparticles would be a suitable drug delivery system for anticancer drug delivery to over MDR, in cancer.

## 2. Materials and Methods

### 2.1. Materials and Animals

Paclitaxel was obtained from the Tianfeng Bioengineering Technology Co., Ltd. (Shenyang, China). Vitamin E and bovine serum albumin was obtained from Sigma-Aldrich (St. Louis, MO, USA). Cremophor EL was obtained from BASF Corporation (Ludwigshafen, Germany). 3-(4,5-Dimethyl-thiazol-2-yl)-2,5-diphenyl-tetrazolium bromide (MTT) was obtained from Sigma (St. Louis, MO, USA). RPMI-1640 and fetal bovine serum were obtained from Gibco (BRL, Gaithersburg, MD, USA). All other chemicals and solvents were of analytical or chromatographic grade and were used without further purification.

The PTX solution was prepared according to the clinical formulation. In brief, PTX (0.012 g) was dissolved in anhydrous ethanol (1 mL) and Cremophor EL (1 mL), under magnetic stirring. PTX solution was diluted with saline, before the test.

Balb/c mice (16–18 g) were obtained from the Experimental Animal Center (Nantong University, China). All animal experiments were approved by Nantong University Ethics Committee (20180512-001) and conformed to the Guidelines for the Use of Laboratory Animals.

### 2.2. Preparation of PTX NPs and PTX-VE NPs

The PTX NPs and PTX-VE NPs were fabricated by a desolvation-ultrasonication technique, as described in our previous report, with some modifications [29]. In brief, BSA (0.20 g) was dissolved in 5 mL deionized water, with magnetic stirring. VE (0.01 g) and PTX (0.01 g) were dissolved in 0.3 mL anhydrous ethanol. Then, the VE and PTX mixed solution were added dropwise to the BSA solution, with magnetic stirring. The mixtures were dispersed by probe ultrasonication (JY92-II, Ningbo Scientz Biotechnology Co., Ltd., Ningbo, China) at 400 W, for 4 min, in an ice bath with a 3 s pulse-on period and a 1 s pulse-off period. After sonication, anhydrous ethanol was evaporated by a rotator RE-2000 (Ya Rong Biochemical Instrument Factory, Shanghai, China). Subsequently, the samples were centrifuged at 3000 rpm, for 10 min, to remove the unloaded drug and impurities, and passed through a 0.45 μm filter membrane for removing the larger particles. The obtained suspensions were kept at 4 °C.

### 2.3. Characterization of PTX-VE NPs

#### 2.3.1. Size Distribution and Morphology

The particle size and polydispersity index (P.I.) of the NPs was determined by dynamic light scattering (PSS NICOMP 380, Santa Barbara, CA, USA). The morphology of NPs was evaluated by transmission electron microscopy (TEM) (JEOL, Tokyo, Japan). In brief, the samples were diluted with distilled water and dropped onto a copper grid. The excess sample was removed with filter paper. Then, 2% phosphotungstic acid staining solution was dropped onto the grid. Finally, the sample was air-dried and assessed with TEM.

#### 2.3.2. Determination of Entrapment Efficiency (EE) and Loading Capacity (LC)

The EE and LC were determined by the method described in our previous research [29]. To separate PTX from the PTX-VE NPs, acetonitrile was added to precipitate BSA, via sonication, for 5 min. After centrifugation at 12,000 rpm for 10 min, the PTX concentration in the supernatant was determined by HPLC. The drug encapsulation efficiency (EE) and loading capacity (LC) were calculated as follows:

(1) $$\mathrm{EE}\left(\%\right)=\frac{W_{drug\;in\;NPs}}{W_{total\;drug}}\times 100$$

(2) $$\mathrm{LC}\left(\%\right)=\frac{W_{drug\;in\;NPs}}{W_{excipients\;and\;drug}}\times 100$$

### 2.4. Docking Studies

Geometry of VE was optimized in gas phase. The calculation was conducted using the GAMESS suit of codes with the hybrid functional B3LYP. The 6-31G (dp) basis set was used for all the elements.

Docking studies were executed using Autodock Vina.9 [27]. BSA crystalline protein structure was obtained from protein databank (Bovine serum albumin: 4OR0, http://www.rcsb.org). The structure of VE used for docking studies was optimized with DFT calculations. Protein structures were altered to include polar hydrogen atoms. During docking studies, the protein structure was kept rigid. Rotation in the VE complex and the PTX complex was permitted for all single bonds.

### 2.5. In Vitro Drug Release

The release behavior of PTX from the PTX-VE NPs was evaluated by a dialysis method. The phosphate buffered saline (PBS, pH 7.4) containing 0.5% *w*/*v* Tween 80 was used as the dissolution medium. In brief, the samples were suspended in a flask and then immersed in the dissolution medium at 37 °C under at 120 rpm. The amount of PTX released was determined by HPLC.

### 2.6. Cytotoxicity of PTX-VE NPs

The MCF-7 and MCF-7/ADR cell line were purchased from Nanjing Kaiji Biotech. Ltd. Co. (Nanjing, China). The cells were cultured in RPMI medium, supplemented with 10% FBS and 1% penicillin-streptomycin, at 37 °C, with 5% CO_2_ and 95% relative humidity. The Cells were seeded in 96-well plates, at a density of 5.0 × 10^3^ cells/well. After 48 h incubation, the growth medium was replaced with 200 μL medium containing VE, PTX solution, and PTX-VE NPs with different concentrations, respectively. Then, each well was added with 20 μL MTT (5 mg/mL) solution and was incubated for an additional 4 h. The culture medium was removed and 200 μL DMSO was added to each well to dissolve the formazan. The absorbance at 492 nm was measured in a microplate reader (Model 500, San Francisco, CA, USA). The results were expressed as % cell viability (OD of treated group/OD of control group ×100).

### 2.7. Rhodamine 6G Accumulation in MCF-7/ADR Cells

MCF-7/Adr cells were seeded onto the cover glasses at 1 × 10^5^ cells in 6-well plate, for 24 h. The cells were washed and incubated with Rhodamine 6G solution (Rho solution), Rho NPs, and Rho-VE NPs, at 37 °C, for 2 h. Then the cells were washed twice with 4 °C PBS and fixed with 4% paraformaldehyde, for 20 min. The nuclei were counterstained by 4′,6-diamidino-2-phenylindole (DAPI). The Rho 6G fluorescence was visualized by confocal laser scanning microscope (CLSM, TCS SP2/AOBS, LEICA, Bensheim, Germany).

### 2.8. Therapeutic Efficacy in Resistant Breast Cancer Xenografts Mice

The anti-tumor effect was evaluated in MCF-7/ADR tumor bearing Balb/c mice model. MCF-7/ADR cells were injected subcutaneously, at 2 × 10^7^ cells, in the armpit of the right anterior limb. When the tumor sizes reached about 100 mm^3^, the mice were randomly divided into four groups (n = 6), and the formulations of PTX solution, PTX NPs, and PTX-VE NPs were intravenously administrated at a dose of 10 mg/kg, at two-day intervals, for five times, with physiological saline as control. Tumor volumes and Body weights were measured with a caliper every other day. Tumor volume (*V*) was determined by the following formula:

(3) $$V=\frac{\pi ab^{2}}{6}$$

where a and b represent the long and short axis of tumor, respectively.

### 2.9. Statistical Analysis

Analysis of statistical significance was performed with the SPSS statistics software 16.0. The data are presented as the mean ± SD. Student’s *t*-test was used to analyze the differences. The differences were considered significant at *p* < 0.05.

## 3. Results and Discussion

### 3.1. Preparation and Characterization of PTX-VE NPs

The PTX-VE NPs were fabricated by the desolvation-ultrasonication method. The PTX and VE mixed ethanol solution was added to the BSA solution, and the mixture was subjected to probe sonication. Subsequently, the PTX-VE NPs was formed by the interaction among the PTX, VE, and BSA. Both PTX and VE have high affinity with albumin [34,35], the PTX and VE can bound tightly to BSA via hydrophobic interactions and hydrogen bond, which were in favor of the formation of PTX-VE NPs.

**Figure 1:**
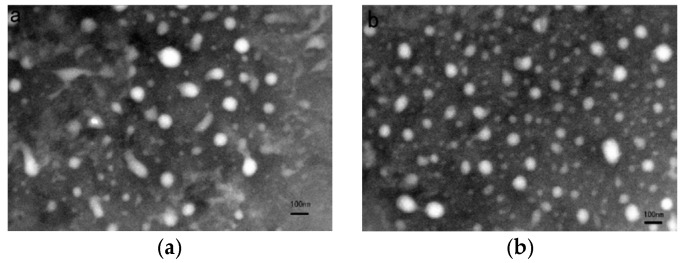
TEM image of PTX NPs (**a**) and PTX-VE NPs (**b**).

The average size of the PTX NPs and PTX-VE NPs was approximately 100 nm (Table 1), which was considered effective for the accumulation of nanoparticles, in tumor tissue, via passive targeting. The PTX encapsulation in the PTX-VE NPs was increased significantly with a loading capacity > 12%, which was five-fold more than that of PTX NPs, owing mainly to the solubility of PTX in the VE oil. The NPs also have a higher drug encapsulation efficiency (>90%). The morphology of the NPs was evaluated by TEM, which showed a uniform and spherical shape (Figure 1).

### 3.2. Docking Studies

**Figure 2:**
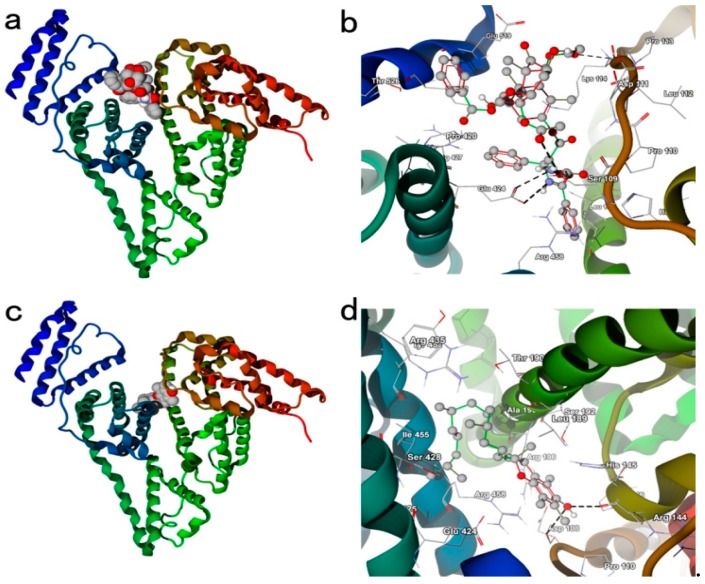
The molecular docking BSA with PTX (**a**,**b**) and VE (**c**,**d**).

Docking studies were performed to investigate the interaction between PTX and BSA, VE and BSA, respectively (Figure 2). The crystalline structure of BSA was obtained from the protein databank. In thermodynamics, a negative binding affinity demonstrates a favorable interaction system. The calculated binding affinity of PTX and VE with BSA were −8.3 kcal/mol and −7.5 kcal/mol, respectively, indicating that PTX and VE have strong interaction with BSA. As shown in Figure 2, there were hydrogen bonds between the Glu 424, Ser 109, Lys 114 of BSA and the nitrogen atom, oxygen atom, oxygen atom of PTX, respectively. There also existed hydrogen bonds between the Arg144, Asp108 and the hydroxy of the VE, respectively. Moreover, hydrophobic interactions were another important interaction between the PTX/VE and the BSA. The hydrophobic interactions were established between the Ile 522, Leu 189, Val 423, Ala 193, and PTX; these interactions also occurred between the Ile 455, Val 425, Leu189, Ala 193, and VE. In addition, the hydrophobic interactions and hydrogen bonds between the VE and the PTX cannot be ignored. In summary, the intermolecular force, such as hydrophobic interactions and hydrogen bonds played significantly important role in the formation of the PTX-VE NPs.

### 3.3. In Vitro Drug Release

**Figure 3:**
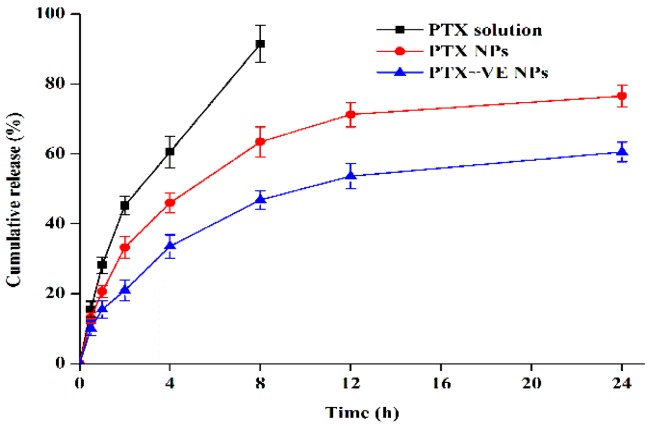
In vitro release profile of PTX from PTX solution, PTX NPs, and PTX-VE NPs, in phosphate buffered saline (0.5% of Tween 80 in PBS, pH 7.4), at 37 ± 0.5 °C (n = 3).

The profiles of the PTX release from the NPs are showed in Figure 3. Nearly 90% of the PTX was released from the PTX solution, in 8 h. Both, PTX NPs and PTX-VE NPs exhibited a sustained release profile, as compared with the PTX solution. After 24 h, the PTX release was about 76% and 60% for the PTX NPs and PTX-VE NPs, respectively. The PTX release from the PTX-VE NPs was slower than that of the PTX NPs. The release of PTX from the NPs might be affected by interactions among PTX, VE, and albumin, such as Hydrogen bonds, π-π stacking, and hydrophobic interactions. In addition, the relative rapid release of PTX from NPs, in the early stage, was likely due to the diffusion of drugs absorbed at the outer shell, while the sustained release in the late stage was probably related to the gradual diffusion of drugs in the inner core of NPs.

### 3.4. Cytotoxicity of PTX-VE NPs

**Figure 4:**
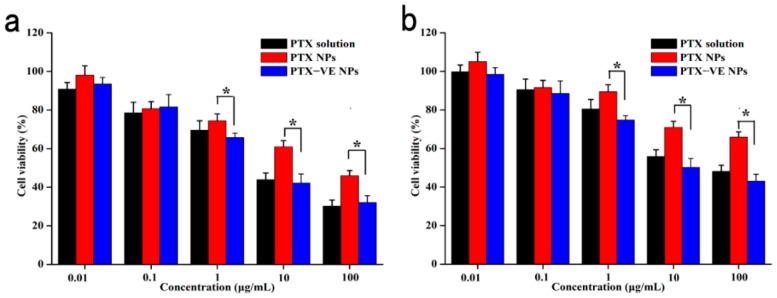
In vitro cytotoxicity of PTX solution, PTX NPs, and PTX-VE NPs, against MCF cells (**a**) and MCF-7/ADR cells (**b**). Data represented the mean ± S.D. (n = 3). * *p* < 0.05, significant difference.

The cytotoxicity study of the PTX solution, PTX NPs, and the PTX-VE NPs was evaluated using the MTT assay and the results are show in Figure 4. It is clear that cell viabilities for all the formulations in MCF-7 cells were lower than that at concentration 1–100 μg/mL in MCF-7/ADR cells, due to P-gp-mediated efflux, which could reduce the drug accumulation in the cells. The PTX-VE NPs exhibited better cytotoxic effect than the PTX NPs, indicating that the PTX-VE NPs could exert stronger MDR-overcoming effects on the MCF-7/ADR cells. In addition, the cytotoxicity of the PTX-VE NPs showed no significant difference, as compared with the PTX solution. The reason was deduced that the cytotoxicity of the PTX solution was partially attributed to the use of Cremophor EL/ethanol mixture, which had 42.1% cytotoxicity on the MCF-7 cells [36]. However, in the case of the PTX-VE NPs, the nanocarriers are biocompatible, and the cytotoxic effect was induced mainly by the PTX incorporated in the nanoparticles [37].

### 3.5. Rhodamine 6G Accumulation in MCF-7/ADR Cells

**Figure 5:**
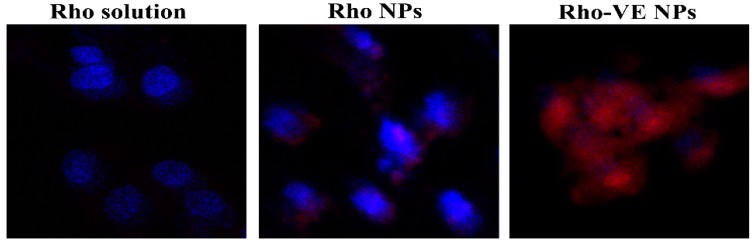
Confocal laser scanning microscope images of various Rhodamine 6G (Rho) labeled formulations in the MCF-7/ADR cells. Original magnification: 10 × 20.

As a substrate of the P-gp efflux pump, the Rhodamine 6G (Rho) was often used as a fluorescent probe to evaluate the whether the improved cytotoxicity of the PTX-VE NPs was due to the increased intracellular drug delivery in MCF-7/ADR cells. Figure 5 shows the fluorescence images in the MCF-7/ADR cells, after 1 h, following the different Rho-labeled formulation treatments. The cells treated with Rho solution exhibited the least amounts of fluorescence signals, which could be attributed to the uptake inhibition of Rho by the P-gp efflux transporter, overexpressed in the MCF-7/ADR cells. Cells treated with the Rho NPs showed increased fluorescence signals, in comparison with the Rho solution. This was consistent with the research that the nanocarriers were able to circumvent the P-gp efflux pump by entering the cells through an endocytic process. However, the increased cell uptake of the Rho NPs was still restricted. The reason inferred was that the rapid release of encapsulated drugs in the cytoplasm was still probably shuttled out of cells, by the P-gp, which is overexpressed on the MCF-7/ADR cell membranes. Furthermore, the strongest fluorescence signals were observed in the cells treated with the Rho-VE NPs. This could be ascribed to the synergic combination of the nanocarrier and the VE. The nanocarrier increased the intracellular drug delivery by circumventing the P-gp efflux pump and the P-gp ATPase activity was probably inhibited by the released VE from the nanoparticles in cells.

### 3.6. Anti-Tumor Effect and Safety in the Xenograft Model

**Figure 6:**
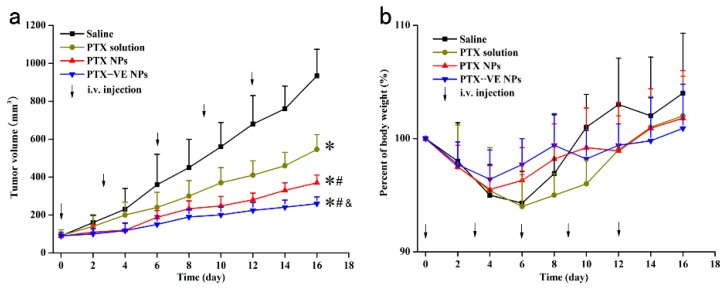
Anti-tumor effect of PTX formulations on the Balb/c mice implanted with MCF-7/ADR cells. Mice were injected through tail vein with Saline, PTX solution, PTX NPs and PTX-VE NPs, at a dosage of 10 mg/kg. The changes of tumor volume (**a**) and body weight (**b**) during 16-day treatments. (n = 6). * *p* < 0.05, significant difference when compared with Saline, ^#^ *p* < 0.05, significant difference when compared with PTX solution. ^&^ *p* < 0.05, significant difference when compared with PTX NPs.

The anti-tumor effect of the PTX-VE NPs was studied in the Balb/c mice bearing the MCF-7/ADR tumor xenograft model. The changes of tumor value and body weight were shown in Figure 6a, the tumor growth rates of different formulations, in ascending order, was PTX-VE NPs, PTX NPs, PTX solution and Saline, respectively. The results indicated that the PTX-VE NPs were the most effective formulation to treat MDR breast cancer. During the treatment, the relative body weight change of mice was also measured, as shown in Figure 6b. No significant body weight loss was observed for the PTX-VE NPs, PTX NPs, PTX solution and Saline, throughout the experiment, demonstrating good safety and low systemic toxicity of these formulations.

**Figure 7:**
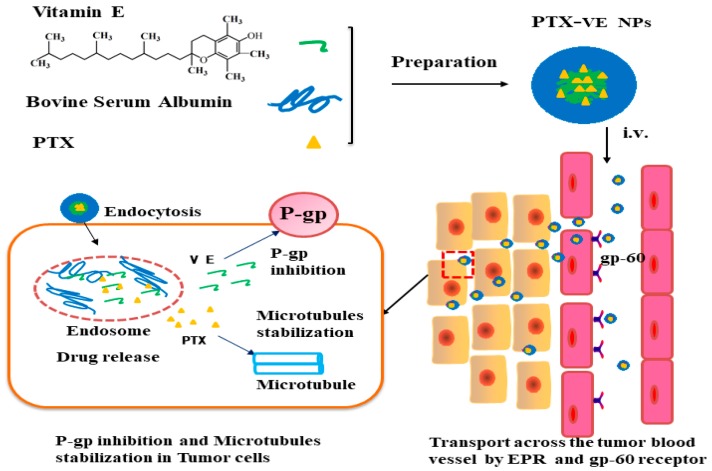
Schematic illustration of the PTX-VE NPs to reversal of MDR.

Accordingly, PTX-VE NPs was a safe and efficient drug delivery system to overcome MDR. The reasons could be deduced to the following aspects, as shown in Figure 7. First, the PTX-VE NPs with particle size less than 200nm, which was able to avoid the rapid reticuloendothelial system uptake and achieved a high accumulation in the tumor site by an enhanced permeability and retention (EPR) effect [35]. Second, the NPs could utilize the albumin receptor (gp60) mediated endocytosis through the blood vessel endothelial cells in the angiogenic tumor vasculature and an increased intratumoral accumulation [10,26]. Finally, the combination of the PTX and the VE in the NPs, would significantly enhance the intracellular drug uptake efficiency in MDR tumor cells and improve the therapeutic efficiency of resistant cancers.

## 4. Conclusions

The present study fabricated the PTX loaded VE-Albumin core-shell nanoparticles to treat MDR breast cancer. The fabricated nanoparticles showed satisfying particle size, drug loading, and sustained-release profile, in vitro. Compared with PTX NPs, PTX-VE NPs exhibited higher cellular uptake and stronger cytotoxic effect in MDR breast cancer cells. Therefore, the PTX loaded VE-Albumin core-shell nanoparticles could be a potential strategy for the treatment of MDR breast cancer.

## Figures and Tables

**Table 1:** Physicochemical characteristics of PTX NPs and PTX-VE NPs (n = 3).

Formulation	Size (nm)	P.I.	Zeta Potential (mV)	EE (%)	LC (%)
PTX NPs	101.2 ± 2.8	0.167 ± 0.03	−2.15 ± 0.6	91.2 ± 3.0	2.5 ± 0.08
PTX-VE NPs	106.9 ± 3.2	0.172 ± 0.02	−20.64 ± 0.8	95.7 ± 2.1	12.5 ± 0.15
